# Diaphragm Function in Very Preterm Infants at 36 Weeks' Postmenstrual Age

**DOI:** 10.1002/ppul.71121

**Published:** 2025-05-12

**Authors:** Benjamin Stoecklin, Zeena Al‐Obaidi, Jenny Svedenkrans, Raffaele Dellacà, J. Jane Pillow

**Affiliations:** ^1^ School of Human Sciences University of Western Australia Perth Western Australia Australia; ^2^ Department of Neonatology University Children's Hospital Basel (UKBB) Basel Switzerland; ^3^ Medical School University of Western Australia Perth Western Australia Australia; ^4^ Department of Clinical Science, Intervention and Technology, Division of Pediatrics Karolinska Institutet Stockholm Sweden; ^5^ Department of Neonatology Karolinska University Hospital Stockholm Sweden; ^6^ Dipartimento di Elettronica, Informazione e Bioingegneria—DEIB Politecnico di Milano University Milan Italy; ^7^ Wal‐yan Respiratory Research Centre Telethon Kids Institute Perth Western Australia

**Keywords:** bronchopulmonary dysplasia, prematurity, respiratory muscle function

## Abstract

**Objectives:**

Understand how bronchopulmonary dysplasia (BPD) and antenatal and postnatal factors influence diaphragmatic functional effectiveness in very preterm infants.

**Working Hypothesis:**

Diaphragmatic functional effectiveness during spontaneous breathing is impaired in infants with BPD. Moreover, diaphragmatic functional effectiveness is influenced by adverse antenatal and postnatal factors.

**Methodology:**

Diaphragmatic functional effectiveness was assessed in a single‐centre, prospective observational study in preterm infants. Transdiaphragmatic pressure (Pdi) and respiratory flow were measured during quiet sleep at 36 weeks' postmenstrual age (PMA). Pdi was normalized to tidal volume (Pdi/*V*_T_). Diaphragmatic work of breathing per minute was calculated from the inspiratory pressure time integral (PTIdi) and respiratory rate. Factors predictive for each outcome were identified from multivariable linear regression.

**Results:**

Very preterm infants (*n* = 182) were measured at a median (IQR) 35.6 (1.3) weeks' PMA. Infants with BPD had a lower Pdi/*V*_T_ (*p* = 0.007) and lower PTIdi·min^−1^ (*p* = 0.022) but higher minute ventilation (*p* = 0.032) and similar respiratory rates (*p* = 0.419) compared to infants without BPD. Birthweight *Z* score (*R* ^2^ = 0.08, *p* < 0.001) and BPD (*R* ^2^ = 0.04, *p* = 0.022) were independent negative predictors for Pdi/*V*_T_ while gestational age (*R* ^2^ = 0.04, *p* = 0.01) and average early postnatal energy intake (*R* ^2^ = 0.03, *p* = 0.026) were independent positive predictors for PTIdi·min^−1^ on multivariable analysis. Chorioamnionitis and duration of mechanical ventilation did not contribute to the final model.

**Conclusions:**

Contrary to our hypothesis, diaphragm functional effectiveness appears improved in infants with BPD. We speculate this finding may reflect an adaptive process, or alternatively indicate an increased recruitment of accessory muscles to achieve required ventilation in BPD infants. Adverse antenatal and postnatal factors only explain a small proportion of variance in diaphragm effectiveness.

## 1 Introduction

The diaphragm is the primary respiratory muscle responsible for generating and maintaining the force needed for sufficient pulmonary gas exchange [1, 2]. Diaphragm dysfunction is one of the suspected causes for respiratory failure in preterm infants [3]. Current understanding of how antenatal and postnatal factors contribute to diaphragmatic dysfunction mostly arises from animal models. These factors include the relatively hyperoxic environment compared to the hypoxic in utero environment, infections, chronic inflammation, poor intrauterine and postnatal growth, corticosteroid therapy and mechanical ventilation [2, 4, 5, 6, 7, 8, 9, 10, 11]. Mechanical ventilation, although life‐saving, promotes ventilator‐induced diaphragmatic dysfunction (VIDD) characterised by muscle atrophy and myofibril injury [12, 13, 14]. VIDD is described in ventilated animals, and in ventilated adults and children [12, 14, 15, 16]. In premature born infants, duration of mechanical ventilation and gestational age are the key predictors for the severity of bronchopulmonary dysplasia (BPD) [17, 18]. Likewise, mechanical ventilation and gestational age play a key role in VIDD [12]. Therefore, logical extension of these findings suggests that infants with BPD may have impaired diaphragmatic functional effectiveness.

The preterm infant diaphragm has fewer slow‐acting type I muscle term fibres compared to the diaphragm of healthy term counterparts [4]. The slow‐acting type I muscle fibres prevent fatigue, resulting in better tolerance of respiratory loads [3, 19]. Type I muscle fibres increase with postmenstrual age (PMA), resulting in an increased type I to type II muscle fibre ratio, with advancing postnatal maturation [3]. Moreover, the immature muscle is comprised predominantly of type IIA fibres, which are fast oxidative and relatively fatigue resistant fibres, whereas the fatiguable type IIX typically appear post‐term [20]. Consequently, the different combinations of muscle fibres in the preterm infants leads to a weaker but at the same time a more fatigue resistant diaphragm [4].

Few studies report on the effectiveness of diaphragmatic function in premature infants, and existing studies have low sample sizes [21, 22, 23]. No reported studies systematically analysed the contribution of antenatal and postnatal factors to diaphragmatic functional effectiveness in very premature infants before discharge home. Moreover, data comparing diaphragmatic functional effectiveness in infants with and without BPD are scarce.

The effectiveness of diaphragmatic function in infants can be assessed with different methods. Measurements during tidal breathing, spontaneous crying or with phrenic nerve stimulation are described in the literature [21, 22]. Key variables of interest are contractile force generated by the diaphragm, and diaphragmatic work of breathing. Work of breathing is determined by the pressure and volume characteristics of the respiratory system. The work performed by the diaphragm and the accessory respiratory muscles must be sufficient to overcome the elastic recoil of the chest wall and the tendency of the lungs to collapse. Moreover, the force generated by the respiratory muscle is needed to overcome the resistance to airflow. Work of breathing increases with increased resistance to airflow, decreased compliance or with increased respiratory rate (RR) [24]. We would therefore expect higher work of breathing in sicker and/or more premature infants if the distribution of work between the diaphragm and accessory muscles is the same.

We aimed to measure tidal volume (*V*_T_) and transdiaphragmatic pressure (Pdi), to understand the effectiveness of contractile force (Pdi normalized to tidal volume) and the diaphragmatic work of breathing. Pdi and the diaphragmatic work were assessed during tidal breathing in unsedated sleeping very preterm infants with and without BPD at 36 weeks PMA.

We hypothesised that infants with BPD would have impaired diaphragmatic functional effectiveness. Moreover, we hypothesised that intrauterine growth, gestational age, postnatal nutrition and duration of respiratory support are predictors of diaphragmatic functional effectiveness in very preterm infants at 36 weeks PMA.

## 2 Materials and Methods

### 2.1 Study Design

We designed a prospective observational study to characterise diaphragmatic functional effectiveness defined as diaphragmatic force (Pdi/*V*_T_) and diaphragmatic work of breathing per minute (PTIdi·min^−1^) in unsedated, very preterm infants. The study was approved by the Women and Newborn Health Service Human Research Ethics Committee (HREC:1883EW and 20130193EW) and the University of Western Australia (RA/3/1/5942) in Perth, Western Australia.

### 2.2 Study Infants and Recruitment

Very preterm infants were recruited from the Neonatal Critical Care Unit at King Edward Memorial Hospital for Women (KEMH) in Perth. Informed consent was obtained from the parents before enrolment in the study.

**Figure 1:**
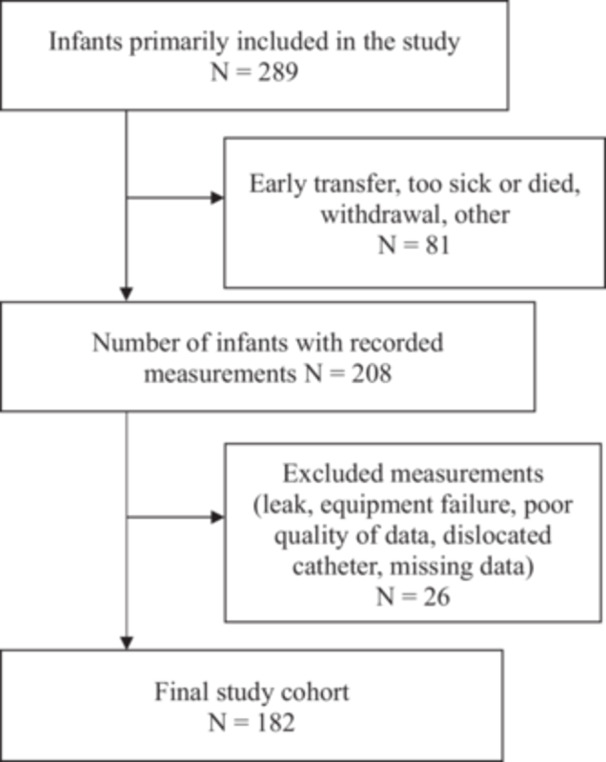
Study flowchart showing all recruited infants and final number of infants with valid measurements.

Infants eligible for the study were born between 21st of July 2013 and 27th of January 2017 at <32 weeks' gestation. All included infants were part of the Preterm Infant Functional and Clinical Outcomes (PIFCO) study (ACTRN126130010627181). Exclusion criteria were major congenital malformations and no parental consent. The recruitment and study enrolment process are shown in Figure 1. BPD was prospectively defined as oxygen dependency > 28 days according to the NIH definition published in 2001 [25].

Study data were collected and managed using Research Electronic Data Capture (REDCap) software hosted at The University of Western Australia. REDCap is a secure, web‐based application designed to support data capture for research studies [26].

### 2.3 Measurement of Breathing Pattern and Diaphragm Function

Measurements were obtained at 36 weeks' PMA in unsedated infants assessed at least 30 min after the last feed, in a supine position, and during behaviourally determined quiet sleep [27].

Airflow (mL/s) was measured using an ultrasonic flow meter (Exhalyser D, EcoMedics, Duernten, Switzerland) attached to a silicone mask. The ultrasonic flow meter was calibrated against the flow generated during 20 mL volume excursions obtained at a rate of approximately 60 excursions/min using a calibration syringe (5510 Series, Hans Rudolph Inc, USA). Tidal volume was derived from the integral of the flow waveform. RR was determined from the number of breaths/minute.

Pdi was recorded using a 2 F (0.67 mm) dual tipped pressure transducer (Mikro‐Tip Transducer, Millar Instrumental Inc. Houston, Texas/USA) with the pressure sensors spaced five centimetres apart. The distal pressure sensor was placed in the stomach and the proximal pressure sensor in the oesophagus. The correct position of the catheter was confirmed in real‐time by opposing phases of the distal and proximal pressure transducer pressures during catheter placement. The pressure signals generated were recorded using the Powerlab data acquisition system and displayed in real time on LabChart Pro software (v7, ADInstruments). Forced manoeuvres and phrenic nerve stimulation were not used.

Pdi was determined as the average waveform amplitude from a minimum of 10 breaths during tidal breathing. Pdi was normalised to tidal volume to provide a relative measure of the effectiveness of diaphragmatic force (Pdi/*V*_T_). The product of the RR and the average breath‐to‐breath pressure time integral of the inspiratory transdiaphragmatic pressure (PTIdi) was calculated as a proxy for the inspiratory diaphragmatic work performed each minute (PTIdi·min^−1^). PTIdi is a measure of the diaphragmatic work of breathing: higher values reflecting a higher work of breathing and lower values a lower work of breathing.

### 2.4 Statistical Analysis

Data were analysed within SPSS (v25·0·0·0; IBM Corp, USA). Parametric data are presented as mean ± standard deviation (SD), non‐parametric data as median and interquartile range (IQR). Diaphragm functional effectiveness in infants with BPD and without BPD was compared using Mann−Whitney *U* test. Potential predictive antenatal and postnatal factors were assessed for collinearity using variance inflation factor. Factors collinear with gestation were regressed against gestation, and the unstandardized residuals were used as the independent effect of each exposure. Univariate analysis was performed to determine which of these antenatal and postnatal factors were associated with diaphragm functional effectiveness. Factors significantly associated with the outcome variables (Pdi/*V*_T_ and PTIdi·min^−1^; linear regression analyses without normalisation of Pdi and PTIdi are reported in the supplemental tables), were included in a stepwise linear regression to identify the key independent predictors of effectiveness of diaphragmatic force, and diaphragmatic work of breathing per minute. A *p* < 0.05 was considered statistically significant.

## 3 Results

Diaphragm functional effectiveness was assessed in 182 out of 289 enrolled infants (Figure 1). The infants were born at a median (IQR) gestational age 28.3 (3.4) weeks' and were measured at a median (IQR) 35.6 (1.3) weeks' PMA. Patient characteristics are shown in Table 1. Descriptive statistics for breathing pattern and diaphragmatic functional effectiveness are shown in Table 2.

**Table 1:** Perinatal characteristics of all measured infants.

	All measured infants	No BPD	BPD	*p* value (95% CI), *χ* ^2^
	*n* = 182	*n* = 121	*n* = 61	
Male	110 (60.4)	73 (60.3)	37 (60.6)	0.966 (0.002)
Gestational age (week)^a^	28.3 (3.4)	29.3 (2.7)	25.1 (2.6)	**<0.01** (−4.15, 3.00)
Birth weight (g)^a^	1050 (501)	1170 (483)	800 (258)	**<0.01** (−464, −300)
Birth weight *z*‐score	0.04 ± 0.85	−0.05 ± 0.81	0.22 ± 0.89	**0.039** (0.01, 0.53)
Postmenstrual age at test (week)^a^	35.6 (1.28)	35.1 (1.1)	36.1 (1.2)	**<0.01** (−0.71, 1.28)
Weight at test (g)^a^	2306 (666)	2215 (501)	2622 (497)	**<0.01** (280, 528)
Weight *z*‐score at test	−0.69 ± 0.92	−0.80 ± 0.91	−0.46 ± 0.91	**0.026** (−0.04, 0.6)
Length at test (cm)	44.2 ± 2.2	44.0 ± 2.2	44.7 ± 2.1	**0.047** (0.01, 1.38)
Length *z*‐score at test	−0.95 ± 0.88	−0.88 ± 0.91	−1.13 ± 0.8	0.079 (−0.51, 0.03)
Average energy intake 1st 28 day (kcal/kg/day)^a^	110 (20)	114 (12)	96 (17)	**<0.01** (12.4, 19.9)
Average protein intake 1st 28 day (g/kg/day)^a^	3.35 (0.46)	3.42 (0.36)	3.16 (0.36)	<0.01 (0.14,0.33)
Any maternal steroids	179 (97.3)	117 (96.7)	60 (98.4)	0.516 (0.422)
Chorioamnionitis	97 (52.7)	56 (46.3)	40 (65.7)	**0.014** (6.065)
Mechanical ventilation (day)^a^	1.0 (4.0)	0.0 (1.0)	12.0 (28.0)	**<0.01** (6.0, 16.0)
Non‐invasive ventilation (day)^a^	38.8 (52.0)	14.8 (38.0)	66.0 (22.5)	**<0.01** (38, 51)
Supplemental oxygen (day)^a^	3.2 (46)	0.4 (3.1)	67.0 (55.9)	**<0.01** (56, 76)
Any surfactant	131 (72.0)	70 (57.9)	61 (100)	**<0.01** (35.72)
Any postnatal steroids	8 (4.4)	0 (0.0)	8 (14.3)	**<0.01** (16.60)

*Note:* Data are shown as mean ± SD. *p*‐values < 0.05 are in bold.

Abbreviations: BPD, bronchopulmonary dysplasia; cm, centimetres; d, days; g, gram; w, weeks; *χ* ^2^, Chi‐squares.

a Median (IQR) or *n* (%).

**Table 2:** Lung function variables for infants with and without BPD.

	All infants (*n* = 182)	No BPD (*n* = 121)	BPD (*n* = 61)	*U*	*p*
*V*_T_ (mL)	15.0 (4.0)	15.0 (4.0)	16.0 (4.5)	4749	**0.002**
*V*_T_/weight (mL/kg)	6.5 (1.7)	6.6 (1.7)	6.2 (1.4)	2996	**0.038**
RR	69 (23)	70 (24)	66 (23)	3420	0.419
V_E_ (mL/min)	1023 (352)	1018 (321)	1173 (360)	4410	**0.032**
V_E_/weight (mL/kg/min)	445 (158)	460 (156)	417 (122)	2901	**0.019**
Pdi (cmH_2_0)	20.8 (7.0)	20.7 (6.6)	21.1 (7.9)	3595	0.776
Pdi/V_T_ (cmH_2_O·mL^−1^)	1.4 (0.5)	1.5 (0.5)	1.3 (0.4)	2794	**0.007**
PTIdi (cmH_2_O·s)	3.9 (2.2)	4.0 (2.2)	3.7 (2.2)	3254	0.193
PTIdi per minute (cmH_2_O·s·min^−1^)	271 (100)	280 (95)	261 (106)	2921	**0.022**

*Note:* Values are median (IQR) with statistical significance determined by Mann−Whitney‐*U*. *p*‐values < 0.05 are in bold.

Abbreviations: AU. arbitrary units; FRC, functional residual capacity; RR, respiratory rate; *V*_*E*,_ minute ventilation; *V*_T_, tidal volume.

**Figure 2:**
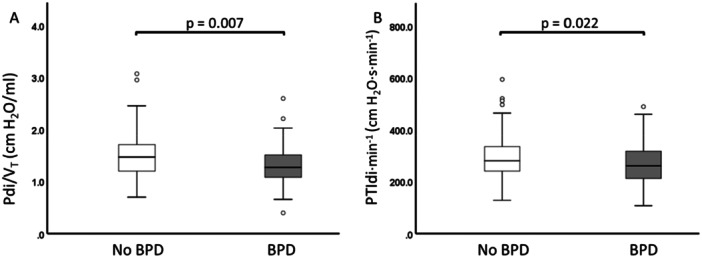
Diaphragm functional effectiveness in infants with and without BPD. (A) Lower Pdi/V_T_ in infants with BPD. (B) Lower PTIdi·min^−1^ in infants with BPD.

*V*_T_/kg increased significantly as gestational age decreased (*R* ^2^ = 0.026, *p* = 0.030). *V*_T_ was significantly higher in infants with BPD (*p* = 0.002), but *V*_T_/kg was lower in infants with BPD compared to infants without BPD. Minute ventilation adjusted for weight was significantly lower in infants with BPD compared to infants without BPD (*p* = 0.019). RR did not correlate with gestational age (*R* ^2^ = 0.009; *p* = 0.210). RR was equal between infants with and without BPD. Infants with BPD had a lower Pdi/*V*_T_ (Figure 2A) and lower PTIdi·min^−1^ compared to infants without BPD (Figure 2B).

### 3.1 Univariate Analysis

Variables related to respiratory support and average energy and protein intake over the first 28 days of life were collinear with gestational age. Therefore, before performing the univariate analysis, we derived the unstandardized residuals for mechanical ventilation, non‐invasive ventilation, duration of respiratory support, duration of supplemental oxygen therapy and average energy and protein intake against gestational age and used the unstandardized residuals as independent measures of respiratory support and oxygen therapy.

Diaphragmatic force (Pdi/*V*_T_) was positively associated with gestational age and negatively associated with weight *z*‐score at birth and a diagnosis of BPD.

Diaphragmatic work of breathing per minute (PTIdi·min^−1^) was positively associated with gestational age and average energy and protein intake over the first 28 days of life, and negatively associated with BPD.

### 3.2 Multivariable Regression

Weight *Z* score at birth and a BPD were significant predictors of Pdi/*V*_T_, and accounted for 10.9% of the variability in Pdi/*V*_T_ (Table 3a). An increase of one weight *Z* score at birth decreased Pdi/*V*_T_ by −0.131 cm H_2_O·mL^−1^ (*R* ^2^ = 0.08, *p* < 0.001). In infants with BPD Pdi/*V*_T_ decreased by −0.184 (*R* ^2^ = 0.04, *p* = 0.022).

**Table 3:** Univariable and multivariable regression.

(3a) Outcome variable: Normalised diaphragmatic contractile force (Pdi/*V*_T_)					
	Univariable regression			Multivariable regression	
				Adjusted *R* ^2^ = 0.119	
	*R* ^2^	*B*	*p*	*B*	*p*
GA (weeks)	0.039	0.036 (0.013)	**0.008**		
Weight *Z* score at birth	0.082	−0.143 (0.036)	**<0.001**	−0.131 (0.036)	**<0.001**
PMA at test (weeks)	0.006	−0.035 (0.033)	0.292		
Avg. energy 1st 28 days (kcal/kg/day)^a^	0.010	0.004 (0.003)	0.170		
Avg. protein 1st 28 days (g/kg/day)^a^	0.015	0.159 (0.098)	0.105		
Chorioamnionitis	0.002	−0.042 (0.063)	0.503		
Maternal steroids	0.000	−0.033 (0.193)	0.865		
Postnatal steroids	0.000	0.031 (0.154)	0.838		
Mechanical ventilation (days)^a^	0.002	0.002 (0.003)	0.575		
Non‐invasive ventilation (days)^a^	0.001	−0.001 (0.002)	0.686		
Oxygen therapy (days)^a^	0.002	0.001 (0.001)	0.568		
Respiratory support (days)^a^	0.000	0.000 (0.002)	0.944		
Any BPD	0.042	−0.184 (0.065)	**0.005**	−0.148 (0.064)	**0.022**
Mod Severe BPD	0.003	−0.061 (0.078)	0.435		

(3b). Outcome variable: Diaphragmatic work of breathing (PTIdi·min^−1^)					
	Univariable regression			Multivariable regression	
				Adjusted *R* ^2^ = 0.06	
	*R* ^2^	*B*	*p*	*B*	*p*
GA (weeks)	0.036	6.877 (2.66)	**0.011**	6.877 (2.63)	**0.010**
Weight *Z* score at birth	0.001	3.849 (7.44)	0.606		
PMA at test (weeks)	0.004	−5.451 (6.56)	0.407		
Avg. energy 1st 28 days (kcal/kg/day)^a^	0.027	1.392 (0.63)	**0.028**	1.392 (0.62)	**0.026**
Avg. protein 1st 28 days (g/kg/day)^a^	0.023	40.15 (19.44)	**0.040**		
Chorioamnionitis	0.001	5.688 (12.62)	0.653		
Maternal steroids	0.000	9.983 (38.54)	0.796		
Postnatal steroids	0.000	−0.182 (30.74)	0.995		
Mechanical ventilation (days)^a^	0.001	−0.231 (0.64)	0.719		
Non‐invasive ventilation (days)^a^	0.000	0.023 (0.38)	0.952		
Oxygen therapy (days)^a^	0.000	−0.018 (0.23)	0.938		
Respiratory support (days)^a^	0.000	−0.054 (0.37)	0.882		
Any BPD	0.032	−32.27 (13.13)	**0.015**		
Mod severe BPD	0.019	−29.19 (15.51)	0.061		

*Note:* p‐values < 0.05 are in bold.

Abbreviations: BPD, bronchopulmonary dysplasia; GA, gestational age; Min, minute; PMA, postmenstrual age; V_T_, tidal volume.

^a^ unstandardized residual versus gestational age.

Gestational age and average energy intake and were significant positive predictors for the inspiratory work of breathing performed by the diaphragm each minute. Gestational age and average energy intake accounted for 6.2% of the variability in PTIdi·min^−1^ (Table 3b). An increase in gestational age by 1 week increased PTIdi·min^−1^ by 6.88 cmH_2_O·s·min^−1^ (*R* ^2^ = 0.036, *p* = 0.01). An increase in one kcal/kg/day over the first 28 days of life increased PTIdi·min^−1^ by 1.39 cmH_2_O·s·min^−1^ (*R* ^2^ = 0.027, *p* = 0.026).

## 4 Discussion

We measured recorded tidal volume, RR and diaphragmatic functional effectiveness during spontaneous breathing and quiet sleep in 182 unsedated very preterm infants with and without BPD at 36 weeks' PMA. We aimed to identify if a diagnosis of BPD was associated with impaired diaphragmatic functional effectiveness, and if specific adverse antenatal factors and postnatal factors were predictive for diaphragmatic functional effectiveness before initial hospital discharge. Contrary to our expectation, infants with BPD used less diaphragmatic force per mL of generated tidal volume (reduced Pdi/*V*_T_) and had lower work of breathing attributable to the diaphragm per minute (PTIdi·min^−1^) than infants without BPD. Whereas both birthweight *Z* score and BPD diagnosis were predictive of Pdi/*V*_T_, these factors only explained 11% of the variability in this outcome variable. Similarly, gestational age and early postnatal caloric intake were predictive perinatal factor associated with PTIdi·min^−1^, but accounted for only 6.0% of the variability in PTIdi·min^−1^. Hence, the variability in these measures of diaphragmatic functional effectiveness during tidal breathing in premature infants remain somewhat unexplained.

We expected that infants with BPD and infants with prolonged duration of respiratory support would need to generate a higher contractile force per unit tidal volume (high Pdi/*V*_T_) and to exhibit increased work of breathing per minute compared to infants without BPD and infants with decreased duration of respiratory support. Similarly, we expected infants born at earlier gestations, and those with reduced caloric intake to also show poor diaphragmatic force and increased diaphragmatic work of breathing. However, infants born more premature, those receiving less caloric intake in the first month of life, and infants with BPD generated lower diaphragmatic forces and lower diaphragmatic work of breathing than their more mature, healthier counterparts in the univariate regression analysis. The reason for this apparent contradiction warrants exploration.

As shown in Table 1, infants with BPD were born more premature, had less caloric intake and were dependent on respiratory support for longer than those without BPD. Infants with BPD had less efficient breathing variables compared to infants without BPD as evidenced by significantly increased tidal volume and minute ventilation. Moreover, weight corrected values for *V*_T_ and minute ventilation were significantly lower in infants with BPD compared to infants without BPD. The absolute and weight corrected values reported for tidal volume in the current study are very similar to those reported by our group in an earlier similar very and extremely preterm infant cohort [28], but lower than those reported by others in less immature preterm infants with and without BPD [29]. A lower weight‐corrected tidal volume and minute ventilation in infants with BPD, directly contradicts our findings on effectiveness of diaphragm force and diaphragmatic work of breathing per minute. A logical explanation for this contradiction is that the diaphragm of infants with BPD has a lower proportional contribution to the driving pressure responsible for overall tidal volume compared to infants without BPD.

The lower Pdi/*V*_T_ and PTIdi·min^−1^ in infants with BPD could be related to a lower chest wall compliance or an increased contribution of force from the accessory respiratory muscles to tidal breathing. We investigated chest wall compliance in another cohort of very preterm infants and found a lower chest wall compliance in infants with moderate and severe BPD compared to infants without BPD [30]. A lower chest wall compliance (stiffer chest wall) may improve diaphragmatic functional effectiveness by reducing chest wall distortion during tidal breathing, and therefore explain the lower Pdi/*V*_T_ and PTIdi·min^−1^ in the more premature infants including those with BPD. Moreover, a decreased Pdi/*V*_T_ and PTIdi·min^−1^ in infants with BPD does not necessarily mean a decrease in the work of breathing as our measurements were confined to assessment of diaphragmatic force and diaphragmatic work of breathing. In infants with BPD, a higher proportion of the *V*_T_ could be generated by the accessory respiratory muscles relative to the contribution from the diaphragm. Therefore, low diaphragmatic force generation and diaphragmatic work of breathing may reflect a poorly functioning diaphragm rather than increased effectiveness. We are unable to confirm this hypothesis, as we have not assessed the muscle activity of the whole respiratory system with simultaneous measurements of the diaphragm and the accessory muscles.

A further explanation of the lower Pdi/*V_T_* (increased diaphragm functional effectiveness) in the BPD group is that the diaphragm of babies with BPD is shortened due to hyperinflation, meaning that the diaphragm muscle is not resting at optimal length, and unable to generate significant inspiratory pressure. Adaptation to this condition is possible via longitudinal atrophy characterized by loss of sarcomeres along the length of the muscle. Longitudinal atrophy allows the optimum fiber length to be reset for a higher lung volume, and thereby maintain maximal force production [31]. Infants in the BPD group had significantly longer exposure to positive pressure respiratory support compared to the non‐BPD group and hyperinflation is a well known characteristic of infants with BPD. The persistence of lower Pdi/V_T_ in the BPD group compared to the non‐BPD group, suggest that such remodelling has not occurred by 36 weeks PMA. Interestingly, Hutten and co‐workers observed increased ramp inspiratory time in infants with BPD [32]. While this finding could be related to increased resistive work of breathing, it could also point to altered length of diaphragm fibres in association with hyperinflation, impacting on contractile properties and ability to generate pressure.

The multivariable analysis aimed to inform on factors that might contribute to any observed differences in diaphragmatic force generation and diaphragm‐associated work of breathing. Our finding that weight *Z* score at birth is a significant negative predictive factor for Pdi/*V*_T_ indicates that diaphragmatic contraction becomes more effective (lower Pdi/V_T_) as adequacy of in utero growth improves. This finding is consistent with previous observation that intrauterine growth restriction is associated with impaired myogenesis in fetal pigs [33]. Similarly, antenatal nutritional deprivation leading to intrauterine growth restriction decreases diaphragm muscle strength in rat pups [6]. Even though diaphragmatic force and diaphragmatic muscle strength are not directly comparable, one could imagine that not only diaphragm muscle strength, but also force is reduced in rats that are deprived of nutrition before birth. The finding that less diaphragmatic force was generated per unit tidal volume in infants with BPD is most likely explained by the lower chest wall compliance in infants with BPD or alternatively indicate a lower relative contribution of the diaphragm to driving pressure due to recruitment of accessory muscles in the BPD group [30].

PTIdi·min^−1^ increased with increasing gestational age and increasing average energy intake over the first month of age. An increase in PTIdi·min^−1^ with increasing gestational age seems counterintuitive at first. However, we found a lower chest wall compliance in a separate cohort of infants with BPD [30] and the infants diagnosed with BPD were born at a lower gestational age. The observed increase in diaphragmatic work of breathing per minute (PTIdi·min^−1^) with increasing caloric intake appears contradictory as well, given that postnatal nutrition deprivation in rats is associated with reduced diaphragm muscle strength [7]. Impaired nutrition has also a negative impact on diaphragmatic function in young patients with cystic fibrosis, women with anorexia, and undernourished adult patients [34, 35, 36]. However, given there were differences in caloric and protein intake between the BPD and no BPD group over the first 28 days of life, this finding may simply reflect differences in diaphragmatic work of breathing between the BPD and no BPD groups.

We could not find any correlation between antenatal and postnatal steroid exposure and diaphragmatic functional effectiveness, most likely due to the fact that >95% of all infants received antenatal steroids and only a minority of infants received postnatal steroids. Moreover, we could not find any correlation between invasive respiratory support and diaphragmatic functional effectiveness at 36 weeks' PMA. Studies in adult animals show clearly that controlled mechanical ventilation leads to VIDD characterised by muscle atrophy and myofibril injury [12, 16, 37]. Similar findings were reported in long‐term ventilated children and in ventilated adults [12, 15]. In contrast to these studies, our preterm infants received synchronised assisted ventilation, which preserves spontaneous respiratory activity and therefore reduces the incidence and severity of VIDD [38]. Animal studies and studies in human adults were performed whilst the animal or the human‐being were still ventilated or had just come off mechanical ventilation [37]. As such, a possible explanation is that VIDD resolves over time. The infants enrolled in our study were off mechanical ventilation for a mean (±SD) duration of 47 (±15) days before the assessment. Furthermore, except for a limited number of infants (*n* = 16), the included infants were off continuous positive airway pressure (CPAP) support for a mean (±SD) duration of 20 (±14) days before testing. Diaphragmatic functional effectiveness was not different between infants on CPAP compared to infants without CPAP.

Our study has some limitations. The diaphragmatic functional effectiveness was measured using a face mask and an ultrasonic flowmeter. We have potentially overestimated diaphragmatic functional effectiveness due to increased resistance when using this equipment compared to infants breathing without additional impedance. However, measurements during tidal breathing without the face mask were highly variable, making a sensible analysis of these data impossible. Despite continuous real‐time recording via LabChart Pro software, we had to exclude six of our recordings due to dislocation of the catheter during the measurements. We excluded another six infants from the analyses due to leak at the face mask. Whereas the leak would probably have minimal effect on diaphragmatic force measurement, Pdi could no longer be accurately normalised to the measured tidal volume.

We chose to normalize Pdi to the generated tidal volume given there was a small but significant difference in the absolute tidal volume between the two groups that may have resulted from the slightly older age and increased weight of the BPD group at time of test. The BPD infants were also relatively heavy compared to their length at the time of study, and hence the difference in absolute tidal volume may have been driven by metabolic factors such as different CO_2_ production (not measured). To properly understand differences in “effectiveness” of the diaphragm pressures we therefore aimed to compare diaphragmatic pressures per unit of air moved during a breath. Table 2 shows that without this normalization, infants with BPD used the same absolute diaphragmatic pressure to generate a higher tidal volume. Similarly, PTI was standardized to the work of breathing over a full minute to account for differences in respiratory patterns between infants with and without BPD that occur on a breath by breath basis. Without this normalization, Table 2 shows that there was no difference in PTIdi between the two groups.

A comparison between the reported animal studies and our study is challenging: animal models assessed maximum diaphragmatic force and muscle fatigue whereas diaphragm function in humans was assessed previously by phrenic nerve stimulation [3, 4, 21, 34, 35]. We investigated diaphragmatic functional effectiveness during spontaneous breathing and did not focus on maximum force or fatigue. Nevertheless, Dassios et al. reported higher PTIdi in infants with BPD compared to infants without BPD when measured at around the same PMA [39]. However, the preterm infants without BPD in the Dassios study were approximately 4 weeks more mature than our no BPD group. Further, the no BPD group in the Dassios study were measured at around 2 weeks postnatal age, whilst our no BPD infants were measured at around 6 weeks postnatal age. A different method was also used to calculate PTIdi as Dassios and colleagues report the tension time index from a maximal inflation whereas the current study evaluated the pressure time integral during quiet breathing. A comparison of the two study populations is therefore difficult. The assessment of maximum force and phrenic nerve stimulation are difficult to perform in preterm infants. Nevertheless, Dimitriou et al. stimulated the phrenic nerve in infants and found a positive correlation between diaphragmatic pressure production and gestational age [21]. We could have missed associations between diaphragmatic functional effectiveness and antenatal and postnatal factors as the differences in diaphragmatic functional effectiveness may only be evident when maximum force is generated.

The large sample size is a major strength of our study. To the best of our knowledge, this is the largest study to investigate diaphragmatic functional effectiveness in very preterm infants at 36 weeks' PMA. Additionally, we measured our infants during quiet sleep without sedation, and diaphragmatic functional effectiveness was assessed over a period of up to 30 stable breaths. All infants were measured with a microtip dual pressure catheter. The dual tip pressure catheter (Mikro‐Tip Transducer, Millar Instrumental Inc. Houston, Texas/USA) and the simultaneous real time recording via LabChart Pro software enabled continuous assessment of the correct position of the catheter.

## 5 Conclusion

In conclusion, we found no evidence that diaphragmatic functional effectiveness is decreased in infants born more premature or in infants with BPD. Either infants with BPD exhibit more efficient diaphragm function or ineffective diaphragmatic function means these infants are dependent on accessory muscles, which we did not assess in this study. Diaphragmatic effectiveness decreased with improved intrauterine growth but decreased with increased postnatal energy intake. Moreover, infants with increased dependency on mechanical ventilation did not show a decrease in diaphragm function, which could be related to the time interval between extubation and the assessment. Overall, even though weight *Z* score at birth and a BPD diagnosis were statistically significant predictors of Pdi/*V*_T_, they only accounted for 12% of the variability in Pdi/*V*_T_ while gestational age and caloric intake accounted for only 6% for the variability in diaphragmatic work of breathing per minute. Diaphragmatic functional effectiveness in very premature infants is likely influenced by additional as yet unknown antenatal and postnatal factors. Future studies should aim to partition the contribution of the accessory muscles and the diaphragm to the tidal volume generated to further understand the key determinants of overall work of breathing in preterm infants with and without BPD. Furthermore, we suggest that future studies relate diaphragm function to a functional measure of the effectiveness of ventilation such as CO_2_ removal, and measure the geometry of the diaphragm to further understand the extent to which differences between groups may be related to adaptive responses in the diaphragm.

## Author Contributions

**Benjamin Stoecklin:** writing – original draft, investigation, formal analysis, writing – review and editing, visualization, data curation, supervision. **Zeena Al‐Obaidi:** writing – original draft, investigation, formal analysis, writing – review and editing, data curation. **Jenny Svedenkrans:** writing – review and editing, investigation, data curation. **Raffaele Dellacà:** writing – review and editing, methodology. **J. Jane Pillow:** conceptualization, writing – review and editing, funding acquisition, supervision, formal analysis, methodology.

## Ethics Statement

The study was approved by the Women and Newborn Health Service Human Research Ethics Committee (HREC:1883EW and 20130193EW) and the University of Western Australia (RA/3/1/5942) in Perth, Western Australia.

## Conflicts of Interest

The authors declare no conflicts of interest.

## Supporting information

Supplemental Tables Regression analyses.

## Data Availability

The data that support the findings of this study are available from the corresponding author upon reasonable request.
