# Supplementary material for: Diaphragm Function in Very Preterm Infants at 36 Weeks' Postmenstrual Age
Source: Pediatr Pulmonol. 2025 May 12;60(5):e71121. doi: 10.1002/ppul.71121 (PMC12068034; doi:10.1002/ppul.71121)
Supplement: Supplementary file 1 — Supplemental Tables Regression analyses. [file PPUL-60-0-s001.docx]

Supplemental tables 1s and 2s.

Multivariable regression analyses with non-normalised Pdi and PTIdi.

| 1s) Outcome variable: Normalised diaphragmatic contractile force (Pdi) | | | | | |
| --- | --- | --- | --- | --- | --- |
|  | Univariable regression | | | Multivariable regression  Adjusted R^2^ = 0.051 | |
|  | R^2^ | B | p | B | p |
| GA (w) | 0.002 | 0.113 (0.177) | 0.523 |  |  |
| Weight Z score at birth | 0.001 | 0.180 (0.487) | 0.712 |  |  |
| PMA at test (w) | 0.002 | 0.228 (0.430) | 0.597 |  |  |
| Avg. energy 1^st^ 28 d (kcal/kg/d)^a^ | 0.029 | 0.075 (0.032) | **0.021** |  |  |
| Avg protein 1^st^ 28 d (g/kg/d)^a^ | 0.051 | 3.780 (1.217) | **0.002** | 3.780 (1.217) | **0.002** |
| Chorioamnionitis | 0.011 | 1.178 (0.822) | 0.154 |  |  |
| Maternal steroids | 0.001 | 1.278 (2.522) | 0.613 |  |  |
| Postnatal steroids | 0.003 | 1.406 (2.010) | 0.485 |  |  |
| Mechanical ventilation (d) ^a^ | 0.000 | 0.011 (0.042) | 0.802 |  |  |
| Non-invasive ventilation (d) ^a^ | 0.001 | 0.008 (0.025) | 0.750 |  |  |
| Oxygen therapy (d) ^a^ | 0.006 | 0.016 (0.015) | 0.307 |  |  |
| Respiratory support (d) ^a^ | 0.001 | 0.011 (0.024) | 0.649 |  |  |
| Any BPD | 0.001 | -0.408 (0.873) | 0.641 |  |  |
| Mod Severe BPD | 0.000 | 0.063 (1.025) | 0.951 |  |  |
| GA, gestational age; w, weeks; PMA, postmenstrual age; d, days; ^a^unstandardized residual vs. gestational age | | | | | |

| 2s). Outcome variable: Diaphragmatic work of breathing (PTIdi) | | | | | | |  |
| --- | --- | --- | --- | --- | --- | --- | --- |
|  | | Univariable regression | | | Multivariable regression  Adjusted R^2^ = 0.044 | |  |
|  | R^2^ | | B | p | B | p | |
| GA (w) | | 0.012 | 0.080 (0.053) | 0.134 |  |  | |
| Weight Z score at birth | | 0.004 | 0.127 (0.148) | 0.392 |  |  | |
| PMA at test (w) | | 0.000 | -0.017 (0.130) | 0.897 |  |  | |
| Avg. energy 1^st^ 28 d (kcal/kg/d)^a^ | | 0.044 | 0.028 (0.010) | **0.004** | 0.028 (0.01) | **0.004** | |
| Avg protein 1^st^ 28 d (g/kg/d)^a^ | | 0.027 | 0.828 (0.374) | **0.028** |  |  | |
| Chorioamnionitis | | 0.010 | 0.338 (0.249) | 0.177 |  |  | |
| Maternal steroids | | 0.002 | 0.431 (0.765) | 0.573 |  |  | |
| Postnatal steroids | | 0.002 | -0.363 (0.610) | 0.552 |  |  | |
| Mechanical ventilation (d) ^a^ | | 0.002 | -0.007 (0.013) | 0.591 |  |  | |
| Non-invasive ventilation (d) ^a^ | | 0.009 | -0.010 (0.007) | 0.196 |  |  | |
| Oxygen therapy (d) ^a^ | | 0.003 | -0.003 (0.005) | 0.490 |  |  | |
| Respiratory support (d) ^a^ | | 0.014 | -0.011 (0.007) | 0.118 |  |  | |
| Any BPD | | 0.014 | -0.423 (0.263) | 0.110 |  |  | |
| Mod Severe BPD | | 0.024 | -0.652 (0.307) | **0.035** |  |  | |
| GA, gestational age; w, weeks; PMA, postmenstrual age; d, days; ^a^unstandardized residual vs. gestational age | | | | | | |  |
